# Potentio-tunable FET sensor having a redox-polarizable single electrode for the implementation of a wearable, continuous multi-analyte monitoring device

**DOI:** 10.1007/s00216-022-03911-0

**Published:** 2022-02-01

**Authors:** Sharon Lefler, Berta Ben-Shachar, Hila Masasa, David Schreiber, Idan Tamir

**Affiliations:** Qulab Medical, Herzliya, Israel

**Keywords:** Field-effect transistor (FET), Wearable, Biosensors, Electrochemical, Continuous glucose monitor (CGM), Potentiometric

## Abstract

The emerging field of wearable devices for monitoring bioanalytes calls for the miniaturization of biochemical sensors. The only commercially available electrochemical wearable monitoring medical devices for bioanalytes are the amperometric continuous glucose monitoring (CGM) systems. The use of such amperometric methods to monitor glucose levels requires a relatively large electrode surface area for sufficient redox species collection, allowing accurate measurements to be made. Consequently, miniaturization of such sensors bearing large electrodes is challenging. Furthermore, it is difficult to introduce and deploy more than one electrode–based sensor per device, thereby limiting the number of analytes that can be monitored in parallel. To address these limitations, we have employed a non-referenced, single polarizable electrode coupled to a fin-shaped field-effect transistor (Fin-FET). We have discovered that by passivating the FET area by a relatively thick oxide and/or polytetrafluoroethylene (PTFE) polymer, leaving only the polarizable working electrode (WE) exposed, we can monitor redox analytes at the micromolar to millimolar concentration range. We attribute this effect to the WE polarization by the solution redox species. We have exploited the superior sensitivity of the adjacent silicon-based Fin-FET to detect changes in sensor electrode potentials induced by the redox species. Furthermore, we demonstrated the correlation between a specific analyte and the biasing WE potential on the accumulation/depletion of the coupled Fin-FET channel as manifested by the transistor source-drain current. Moreover, we utilized the analyte-electrode potential interaction, which is analyte-specific, to tune the specificity of the sensor towards an analyte of choice. In addition, we demonstrated the use of a single-electrode potentiometric sweep to assist in identifying the accumulation/depletion as a result of analyte-WE state. Collectively, the tiny potentio-tunable electrochemical sensor (PTEchem sensor) area is ~50 × 50 µm, and dedicated wireless transducer facilitates the use of this sensor for wearable continuous, multi-metabolite monitoring.

**Graphical abstract:**

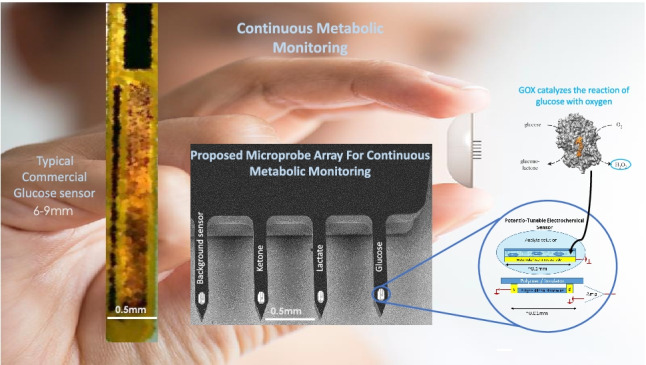

**Supplementary Information:**

The online version contains supplementary material available at 10.1007/s00216-022-03911-0.

## Introduction

The majority of commercial wearable, continuous bioanalytes monitoring devices rely mostly on amperometric techniques [1]. Such techniques typically employ relatively large area surface electrodes for effective electron capture, allowing sufficient current production to overcome the sensor’s electrochemical background noise [2]. Other commercially available technologies rely on fluorescence signals, such as an implantable ~ 2 cm in length device that enables continuous glucose monitoring for up to 6 months. However, implanting this device entails a surgical procedure to position it under the skin (Eversense CGM system, Ascensia) [3]. Other non-invasive technologies rely on spectral analysis [4]. Generally, these types of devices tend to be large and bulky and therefore not suitable for everyday wear [5]. Although many miniaturized redox/electrochemical sensors were demonstrated for lab use [6], and shown to be useful at the single-cell level [7], the majority of these devices require sensitive, sophisticated setup as well as noise shielding systems to reach an acceptable signal-to-noise ratio (SNR). Therefore, such sensors are generally less applicable for use as wearable devices, relying on a simple battery-operated wireless setup.

Alternatively, field-effect transistor (FET)–based biosensors, employing a potentiometric approach for electrochemical signal capture, have been extensively researched for label-free biomolecular sensing for different analytes and sensing applications [8]. These potentiometric sensors benefit from well-established solid-state technologies, such as high sensitivity, miniaturization, low power consumption, and simplicity [9]. They further benefit from integration potential and manufacturing scalability using standard metal oxide semiconductor field-effect transistor (MOSFET) technologies [10]. The ion-sensitive field-effect transistor (ISFET) was invented in the early 1970s [11], employing a designated chemically sensitive field-effect transistor (ChemFET) design [12]. In these FET-based electrochemical sensors, the charge needed to affect the electric field at the gate oxide layer is generated by ions that associate with, or dissociate from, the gate oxide surface [13]. The induced charge in this gate oxide layer changes its surface potential, thereby causing a detectable change of current in the conductive channel of the FET sensor. Generally, ISFETs and ChemFETs monitor changes in pH [14] or dipole, in virtue of the gate oxide layer sensitivity, which can be further modified for the specific interaction of target molecules and analytes [15, 16]. Additionally, most ISFETs and ChemFETs employ a reference electrode [17], limiting their miniaturization potential. Such devices have been shown to detect redox activity via their reactive gate oxide surface [18]. One such sensor type that does not require a reference electrode is the ^E^MOSFET, which detects redox using a redox-reactive iridium oxide gate material [19]. The ^E^MOSFET and other potentiometric solid state–based sensors use a fixed gate potential setup by gate grounding. Gate materials generally rely on reactive substrates to detect such redox species, including different oxides, e.g., silicon oxide and iridium oxide [20, 21], and surface modifiers (e.g., self-assembled monolayers (SAM), quinone, Prussian blue, horseradish proxied (HRP) [22–25], which require specific chemical conditions to control their oxidation states. The use of noble metal as gate electrode building material, on the other hand, should allow for a relatively fast and reversible electrochemical response.

In this paper, we present and characterize a novel sensing paradigm, utilizing a single-electrode electrochemical potentiometric sensor, allowing discrimination between different redox species based on predefined biasing potentials. Specifically, we describe the correlation between the applied biasing potential and the Fin-FET depletion/accumulation effect, which we show to be analyte-specific. This novel sensing paradigm allows reversible redox species sensing realization by optimizing the biasing potential setup.

## Experimental section

### Reagents and chemicals

Recombinant glucose oxidase from *Aspergillus niger*, D/L-glucose, ascorbic acid, caffeine, acetaminophen, creatinine, and hydrogen peroxide were all purchased from Sigma-Aldrich (Merck). Phosphate buffer (150 mM), pH 7.4 was prepared by properly mixing and diluting 1 M monobasic and dibasic potassium phosphate solutions, both purchased from Sigma-Aldrich (Merck).

### Method of PTEchem fabrication

A silicon-on-insulator (SOI) wafer with a buried oxide layer and an ultrathin (55 nm) silicon device layer (SOITEC, France) was employed for PTEchem sensor fabrication. At the end of the process, polymer passivation was added, excluding only the WE, to isolate the Fin-FET both chemically and electrically. Key sensor fabrication steps are illustrated in Figure S1.

### Sensor functionalization

A singulated microprobe chip was further mounted on stainless steel support using an epoxy resin (EPO TEK OE145-5) and wire-bonded to a PCB. Sensor functionalization with glucose oxidase (GOX) was achieved by diluting GOX in a proprietary polyurethane acrylate–based hydrogel containing a commercially available photoinitiator, followed by UV curing. Briefly, the hydrogel formulation was mixed with equal volumes of GOX solution (v/v), reaching a final enzyme concentration of 1U/µL. The resulting GOX-hydrogel was manually deposited on microprobe-carried PTEchem sensors. Hydrogel-coated sensors were dried overnight and UV-cured at 365 nm using commercially available UV illumination equipment (UV-LED Prizmatix).

### Sensing experiments

All sensing experiments were carried out using a custom-designed, Bluetooth (BT)-enabled, 12-channel electronic sampler connected to the sensing chip via wire bonding to a corresponding PCB. This electronic sampler controlled the voltages applied to the WE, source-drain, and back gate (V_WE_, V_SD,_ and V_BG_ correspondingly). Real-time sensing current (*I*_sd_) responses were recorded and graphically presented via dedicated software. Unless otherwise stated, all analyte solutions were prepared in 150 mM phosphate buffer, pH 7.4. Timely and rate-controlled analyte injection was achieved by a Fluigent microfluidics system, comprising Flow-EZ pressure controller, FRP, and ESS PLATFORM, which enable controlling up to 10 different analyte inputs. In experiments imitating skin tissue sensor implantation, the injected analytes passed through an agarose-coated flow cell connected to the microfluidic system (Figure S2). In these experiments, the microprobe-carried sensors were inserted into the agarose layer in contact with the flow cell using a T-LSM050A vertical stage (Zaber).

### Data manipulation

Graph analytics and statistics were performed using GraphPad Prism 9.3.0. Raw data currents (*I*_sd_), where specified, were obtained from the dedicated software and plotted unfiltered in a GraphPad Prism for uniform representation. “Normalized *I*_sd_” data were obtained by dividing the *I*_sd_ recorded from the analyte sample by the *I*_sd_ recorded from the blank sample (phosphate buffer). No further data manipulation or filtering (hardware or software) was employed.

## Results

**Fig. 1:**
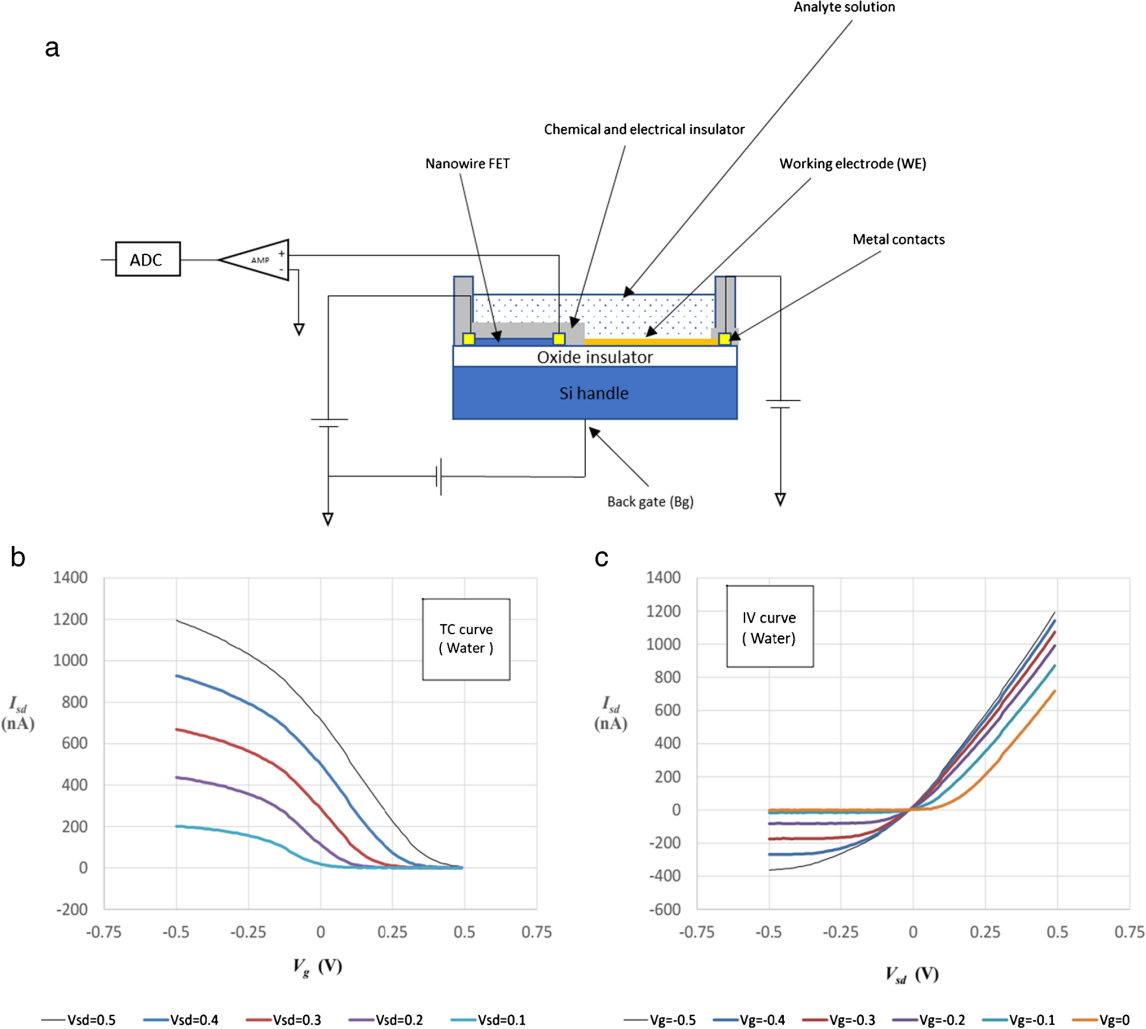
Basic design and general characteristics of a potentio-tunable electrochemical (PTEchem) sensor. **a** schematic representation of the PTEchem sensor structure; **b** and **c** electrical characterization of the PTEchem sensor; **b** TC curves at different *V*_SD_ potentials in distilled water. **c** I/V curves at different WE bias potentials in distilled water

The potentio-tunable electrochemical (PTEchem) sensor setup employs a single electrode (typically, ~ 10 × 50 µm) positioned adjacent to a silicon-based Fin-FET (SiF-FET). This setup reduces the overall sensor size to ~ 50 × 50 µm. Small changes in the working electrode (WE) polarization are amplified by the adjacent Fin-FET, generating charge carrier depletion/accumulation at the FET’s channel, which results in a change of the source-drain current. Figure 1 shows a general schematic setup of the PTEchem sensor. Notably, this setup does not require the use of a reference electrode for sensitive and specific electrochemical sensing. The working electrode (WE) is composed of an inert conductive material, such as a noble metal (gold or platinum), connected to a voltage source that applies a tunable potential to this electrode in reference to the FET’s drain potential. Source and the wafer handle (back gate) terminals are also referenced to the same drain potential.

We have chosen a silicon-fin FET (SiF-FET) architecture design for our MOSFET compatible and scalable PTEchem sensor fabrication process. The design is based on three elements: the SiF-FET channel, WE, and back gate (BG). The WE is connected to a voltage source, which allows applying varying electrode potentials, thereby increasing sensor sensitivity, specificity, and selectivity. Our PTEchem sensor design involves a WE that is fabricated by a top-down approach over a silicon-on-insulator (SOI) wafer substrate (see “2”). To minimize direct chemical interaction between the analyte solution and the SiF-FET through its dielectric surface, we have chemically passivated the surface (excluding the WE) of the PTEchem sensor with polymers such as PTFE or SU-8. This polymer passivation step also minimizes sensor deterioration through device oxide layer corrosion, thereby reducing sensor drift. This results in the WE being the only electrochemically active element exposed to the aqueous solution. As a result, the SiF-FET positioned adjacent to the WE monitors the polarization of the WE, and subsequently not directly involved in analyte sensing. Utilizing this setup, the Fin-FET is capable of detecting minor potential shifts occurring at the WE, displaying high signal-to-noise ratio (SNR). While the typical noise level for this setup is in the range of several nA, the signal levels are measured in hundreds of nA to μA (Figure S3). Figure 1(b–c) depicts typical transconductance and *I*_sd_/*V* curve measurements for a P-type SiF-FET. Measurements were usually performed in DC mode, using defined source, WE, and back gate potentials. Tuning these potentials allows other advantageous features of the PTEchem sensor to be realized, as further detailed and discussed below.

**Fig. 2:**
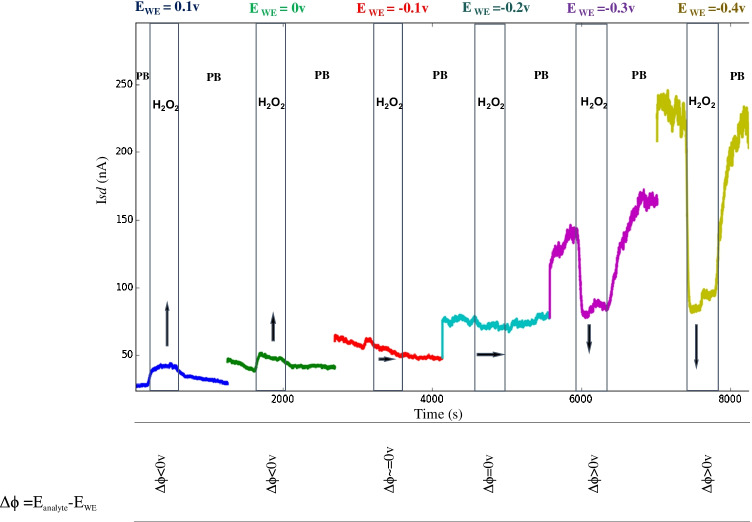
WE potential effect on current (*I*_sd_) direction and amplitude. PTEchem sensor electrical conductance profile in response to hydrogen peroxide (H_2_O_2_ – 1 mM in 150 mM PB) and phosphate buffer (PB). PTEchem sensor at a fixed *V*_SD_ and *V*_BGE_ potentials was subjected to different working electrode (WE) potentials *E*_WE_, ranging from + 0.1 V to − 0.4 V. The resulting *I*_sd_ of a representative PTEchem sensor in response to PB-to-hydrogen peroxide solution exchange was recorded. Arrows indicate current change directions with respect to the PB background

Working at specific WE and SD potentials, the interaction of WE with redox species in a solution resulted in changes to the *I*_sd_. Interestingly, in some cases, the *I*_sd_ output was increased and in others decreased, in response to the same redox species. In order to determine the correlation between *I*_sd_ changes and WE potential, we performed consecutive analyte solution exchanges at different WE potentials. Figure 2 shows the results of a typical experiment performed at different WE potentials, ranging from + 0.1 to − 0.4v. For each applied WE voltage, an exchange from phosphate buffer (PB, 150 mM) to 1 mM H_2_O_2_ (in PB 150 mM solution) was performed sequentially followed by PB reintroduction, while *I*_sd_ was continuously recorded. As depicted, three different *I*_sd_ outcomes were observed in response to the applied WE potential, at constant source and back gate potentials. Taken together, these results suggest that the interaction of redox species, having a specific solution electrochemical potential (*E*_analyte_) with the WE at an initial potential setting (*E*_WE_), induces a galvanic potential change (Δ*ϕ*) at the WE. The overall *E*_WE_ is a combination of source and back gate potentials, both referenced to the drain terminal of the PTEchem sensor. Typically, when the analyte’s redox potential is lower than the applied *E*_WE_ (Δ*ϕ* = *E*_analyte_-*E*_WE_ < 0v), the equilibrium between the solution and the electrode’s overall electric potential will result in negative charging of the working electrode, driven by the transient electron drift from the redox species to the WE, until a new equilibrium is reached. In a P-type semiconductor, this negative charging of the WE leads to an increase in *I*_sd_ (Fig. 2, *V*_WE_ = 0.1v, 0v). Alternatively, when analyte potential and the applied *E*_WE_ are equal (Δ*ϕ* = *E*_analyte_-*E*_WE_ = 0v), the overall electric potential will not change, since no charge is exchanged between the redox species and the WE, resulting in no change to *I*_sd_ (Fig. 2, *E*_WE_ =  − 0.1v, − 0.2v). However, when the analyte redox potential is higher than the applied *E*_WE_ (Δ*ϕ* = *E*_analyte_-*E*_WE_ > 0v), the interaction between the redox species in solution and the electrode’s overall electric potential will lead to transient charge exchange from the WE towards the redox species, effectively resulting in positively charging the WE and thereby leading to reduced I_sd_ in a P-type FET (Fig. 2, *V*_WE_ =  − 0.3v, − 0.4v).

### Analyte redox-specific response

**Fig. 3:**
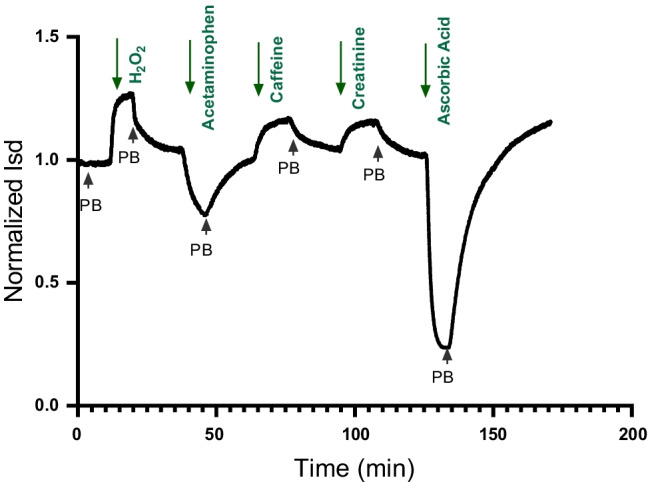
PTEchem response to different redox species. The PTEchem sensor WE was sequentially exposed to H_2_O_2_, acetaminophen, caffeine, creatinine, and ascorbic acid (all at 0.5 mM in 150 mM PB), with phosphate buffer exchange between each analyte, using voltage settings of *V*_SD_ = 0.6; *V*_BGE_ =  − 1.0; and *V*_WE_ =  − 0.4. The *I*_sd_ was monitored continuously and the recorded data is presented as a normalized signal: *I*_sd_ at *t* = *x*/*I*_sd_ at *t* = 0 (phosphate buffer alone)

Next, the effect of different redox species (having the same molar concentration) on the *I*_sd_ at defined *E*_WE_ potentials was measured. Figure 3 shows a representative experiment in which different redox species (all at 0.5 mM in 150 mM PB) were introduced, while WE, back gate, and source potentials of the PTEchem sensor were kept constant. *I*_sd_ was continuously monitored and changes in current direction and amplitude were recorded. Evidently, the sensor’s response to H_2_O_2_, caffeine, and creatinine resulted in *I*_sd_ elevation to different amplitudes, relative to the PB baseline. On the other hand, the interaction of the WE with acetaminophen and ascorbic acid led to a reduction in *I*_sd_, once again displaying the sensor’s response dependence on the identity of the redox species. Hence, it is plausible to conclude that each redox species polarizes the WE according to its characteristic solution redox potential.

### Tunable electrochemical sensor

A major source of electrochemical noise in commercial CGMs results from the presence of interfering redox species (such as acetaminophen, ascorbic acid (vitamin C), hydroxyurea, tetracycline antibiotics, and salicylic acid (aspirin)) in the interstitial fluid (ISF) [26]. These substances may affect CGM performance due to their redox potentials overlapping with the potential applied by the glucose-specific CGM sensor. Consequently, this common source of interference may lead to errors in glucose level monitoring, resulting in compromised CGM performance with poor glycemic control and management.

**Fig. 4:**
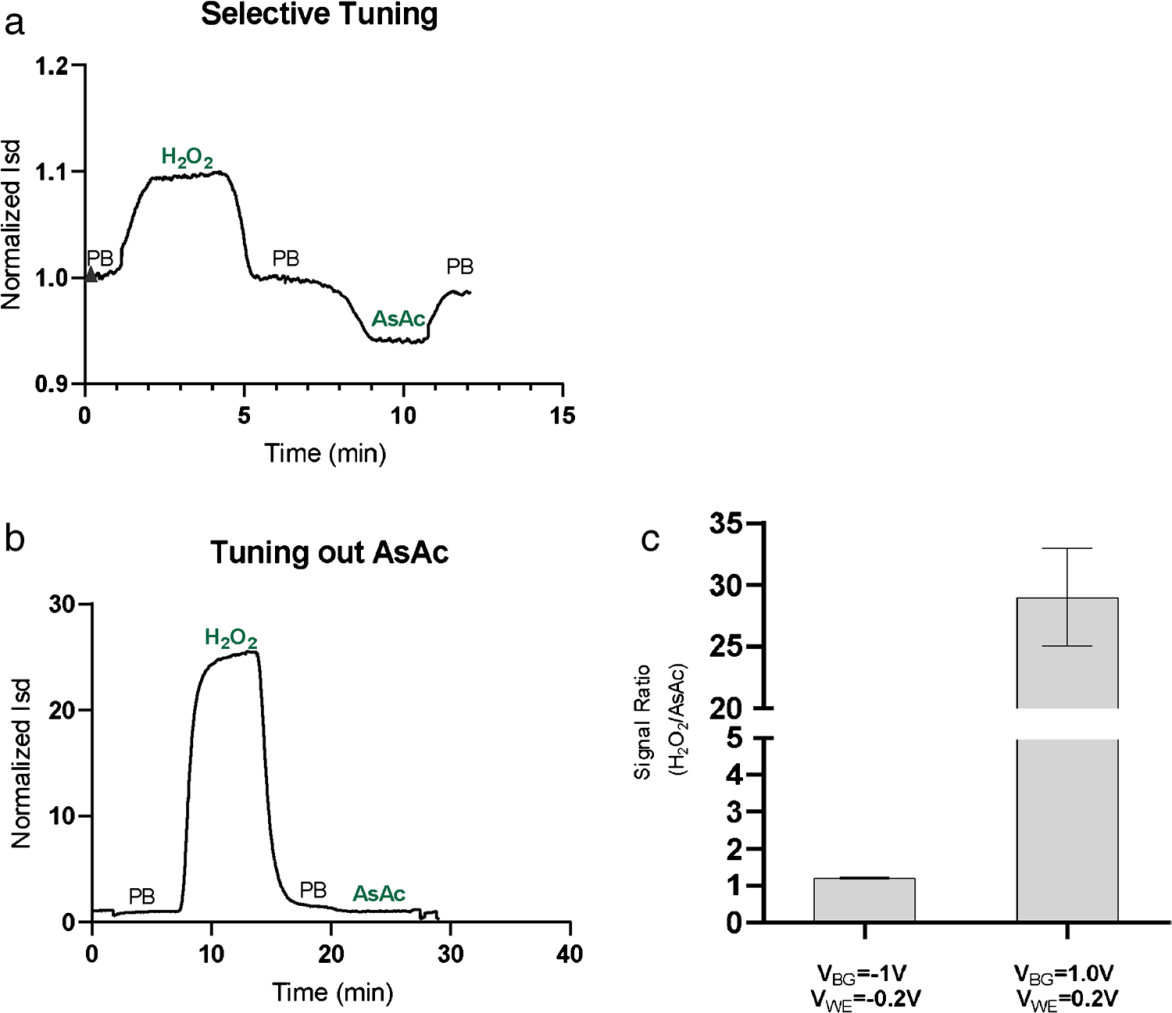
Selectively minimizing PTEchem sensor response to ascorbic acid by tuning WE and BGE sensor biasing potentials. Representative graphs **a** and **b** of continuous sensing over time (*t*) depict a plot of the normalized response signal (*I*_sd_ recorded at (*t*)/*I*_sd_ recorded from baseline PB) of the same device at different voltage settings of the back gate electrode (BGE) and the working electrode (WE). **a** WE exposure to H_2_O_2_ followed by ascorbic acid (AsAc) at potential set to *V*_BGE_ =  − 1 V; *V*_WE_ =  − 0.2 V; **b** tuning the potentials to *V*_BGE_ = 1.0 V and *V*_WE_ = 0.2 V to enhance the signal from hydrogen peroxide, while minimizing the signals in response to ascorbic acid (AsAc). **c** Bar Graph analysis of the results shown in **a** and **b** representing the relative ratio of WE response to H_2_O_2_ vs. ascorbic acid (AsAc)

The ability to control the direction and amplitude of the PTEchem sensor’s response to a specific redox species, combined with the unique redox potential-specific response to a certain analyte, raised the possibility that the sensor’s response could be tuned specifically to monitor a chosen analyte while minimizing its response to interfering ones. To assess this possibility, we tuned the *E*_WE_, by changing back gate and WE potentials, to maximize sensor response to the analyte of interest, while minimizing its response to other substances. Figure 4 presents an in vitro sensing of hydrogen peroxide—a byproduct of glucose oxidase enzymatic catalysis of glucose oxidation, using the PTEchem sensor. After equilibrating the sensor with PB, the sensor was exposed to ascorbic acid—a known interfering substance for CGMs. Both hydrogen peroxide and ascorbic acid elicited a current change (*I*_sd_), however, showing different current directions with respect to baseline (PB) (Fig. 4a and c). By tuning *E*_WE_, a certain WE voltage could be reached in which only a hydrogen peroxide–specific response could be detected (Fig. 4b). As shown in Fig. 4a and b and summarized in Fig. 4c, the electric current elicited in the same sensor that was sequentially introduced to hydrogen peroxide and ascorbic acid solutions was strongly dependent on the voltage settings of the back gate electrode (*V*_BGE_) and the WE (*V*_WE_).

In a sd recording graph presented in Fig. 4a, the PTEchem sensor had the following voltage settings: *V*_BGE_ =  − 1.0v and *V*_WE_ =  − 0.2. At these settings, exposure to hydrogen peroxide led to *I*_sd_ elevation, while the subsequent exposure to ascorbic acid resulted in a decreased current response (relative to the PB baseline). On the other hand, once the voltage of the same sensor was set to *V*_BGE_ = 1.0v and *V*_WE_ = 0.0, the response to hydrogen peroxide resulted in current (*I*_sd_) elevation (Fig. 4b), while the sensor’s subsequent response to ascorbic acid (at the same concentration as used in Fig. 4a) was practically eliminated.

This novel intrinsically tunable property of our PTEchem sensor allows tuning out undesirable electrochemical noise emanating from concurrent redox species. In practical implementation, this feature is expected to reduce or even eliminate the need for stacking semi-permeable membranes on the sensor’s surface, and/or the use of elaborate noise-reducing algorithms for improving CGM specificity. The necessary need for blocking chemical interference consequences with added sensor fabrication complexity and bulkiness with a concomitant increase in sensor price. Importantly, the tunability feature of our PTEchem sensor array should allow assigning different and varying potentials to each of the individual sensors, thereby enabling continuous, parallel, and simultaneous monitoring of multiple different analytes.

### Potentiometry scan and PTEchem sensor calibration

**Fig. 5:**
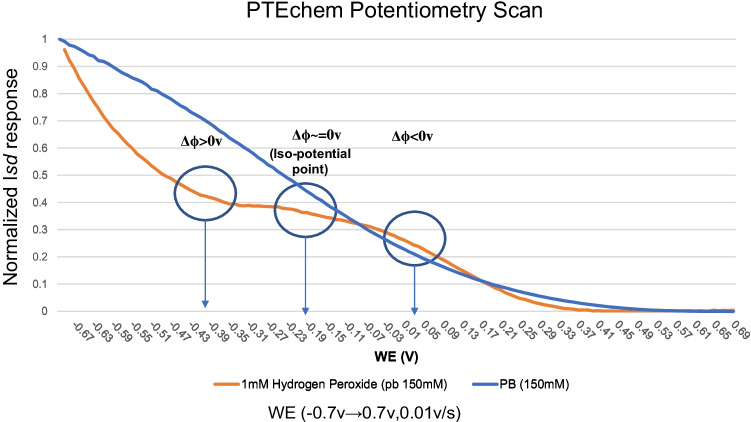
PTEchem sensor potentiometry scan curves of PB solution alone or 1 mM H_2_O_2_ in PB. The change in the drain current was recorded with small incremental changes to the WG potential at constant source and back gate voltage settings in two independent experiments. The first experiment was performed using PB alone (blue line) and the second one with PB containing 1 mM H_2_O_2_ (orange line). The WE potential was changed from − 0.7v to 0.7v at a rate of 0.01v/s. The data presented as normalized *I*_sd_: *I*_sd_ recorded at *V*_WG_ = *x*/*I*_sd_ recorded at *V*_WG_ =  − 0.7v. The polarization peaks were identified by calculating the second and third derivatives of the plot (circled)

As already shown and discussed above, the redox potential of specific analytes differentially affects the direction (accumulation/depletion) and amplitude of the PTEchem sensor response, correlating with the difference (Δ*ϕ*) between the half-cell potential of the redox species and the WE sensor potential bias exerted by the working, back gate, and source electrodes. The graph in Fig. 5 demonstrates how this property could be used for performing potentiometry scan with our PTEchem sensor. This method allows the exploration of the full electrochemical interaction profile between the redox species and the sensor’s electrode. As shown in Fig. 5, the potentiometric scan analysis method reveals redox response peaks representing the maximal negative (Δ*ϕ* = *E*_analyte_-*E*_WE_ > 0v) and positive (Δ*ϕ* = *E*_analyte_-*E*_WE_ < 0v) polarization points, as well as an iso-potential point (Δ*ϕ* = *E*_analyte_-*E*_WE_ = 0v). This behavior is attributed to the WE potential–dependent electrochemical interaction with the analyte, as discussed in the previous section. Accordingly, the iso-potential point is located between the two maximum polarization points, thereby allowing the characterization of different redox analytes and defining their maximal polarization and iso-potential points.

Optimal sensor performance is dependent on the reproducible detection of the target analyte, which is achieved by optimizing the sensor’s sensitivity and specificity. Typically, the PTEchem sensor calibration process will be initiated by identifying the maximal polarization points for the WE potential in the sensor’s response towards a specific analyte. Subsequently, and as shown in Fig. 4 above, the determined analyte-specific iso-potential voltage settings can be used in selectively detecting one analyte over another. Taken together, the calibration process of the PTEchem sensors consists of two steps: first, a “coarse” calibration step, in which the iso-potential point is identified by changing the back gate voltage while keeping the WE voltage constant. This step is followed by a “fine-tuning” calibration, in which the optimal iso-potential point is reached by altering the WE voltage while keeping the BGE constant. Once such an iso-potential point has been determined, an offset voltage is applied to the BGE and WE voltages. This applied offset voltage level and direction are analyte-specific and empirically determined from the PTEchem potentiometry scan curves. For example, in the case of hydrogen peroxide (Fig. 5), a typical working electrode offset from the iso-potential point would range between ± 0.2 and 0.5v, while the back gate offset would range between ± 0.5 and 1v. These offsets to the voltage settings optimize sensor performance robustness, sensitivity, and specificity towards the target analyte.

### Biomedical sensing

**Fig. 6:**
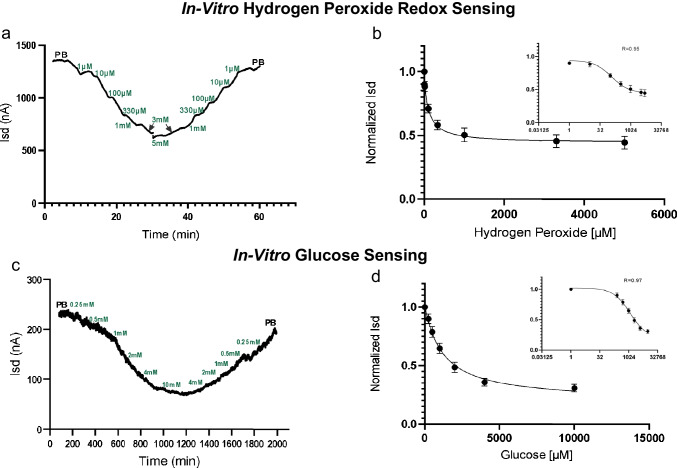
Continuous in vitro sensing of hydrogen peroxide and glucose: **a** Left graph: raw data of *I*_sd_ recorded from one representative PTEchem multi-sensor tuned to optimally measure hydrogen peroxide at a concentration range between 1 and 5000 µM, demonstrating dose-dependent sensor response and reversibility within this concentration range. **b** Right graph: intra-sensor statistical analysis from an array of WEs and their coupled SiF-FETs comprising the multi-sensing PTEchem (depicted in Fig. 1a). The data presented are the mean and standard deviation of *n* = 18 sensors with log2 fit (*R*^2^ = 0.95) for the indicated H_2_O_2_ concentration range. This experiment represents one of > 15 PTEchem batches tested independently. **c** Representative glucose dose–response analysis of microprobe-carried PTEchem sensor, functionalized with GOX. “Normalized *I*_sd_” (Y-axis) was calculated as *I*_sd_-recorded at a corresponding glucose concentration (x-axis) relative to baseline *I*_sd_ (phosphate buffer) which represents the limit of blank (LOB)

To evaluate the feasibility of the PTEchem sensor for potential use in biochemical sensing of physiologically relevant biomarkers, we focused on hydrogen peroxide, a byproduct of glucose oxidation by glucose oxidase. As can be observed in Fig. 6a, changing hydrogen peroxide solution concentrations resulted in a concomitant change of the *I*_sd_. Specifically, increasing hydrogen peroxide concentration resulted in a decrease in *I*_sd_, while decreasing hydrogen peroxide concentration resulted in an increased *I*_sd_. Notably, the sensor displays an in vitro limit of detection of 1 µM for hydrogen peroxide, where the signal is still statistically significant above the blank (± 10nA, Fig. 6, right graph). These results are representative of 15 independently tested PTEchem devices, overall showing a reproducible LOD of < 10 µM. Measured signal-to-noise ratio (SNR) for LOD was extrapolated for equilibrium state sensing and for real-time sensing to be 14.3 and 3, respectively (see supplementary materials S3 for details). Additionally, this experiment also demonstrates the sensor’s reversibility, on changing solutions from low to high analyte concentrations and back, while displaying only minimal sensor drift (Fig. 6a, left graph). The experiment was performed at bias potentials that result in decreasing *I*_sd_ in response to H_2_O_2_ relative to PB. In Fig. 6b, a non-linear regression of normalized *I*_sd_ values at logarithmic fit displays a linear response (*R* = 0.95) and operational concentration range between 10 µM and 3.0 mM hydrogen peroxide.

Next, to establish the transfer function of indirect glucose sensing, we have fabricated a glucose-specific PTEchem sensor by coating the WE surface with a glucose oxidase (GOX)–loaded hydrogel. We have further tested the electrical response of the Gox-functionalized sensor to varying glucose concentrations. The rationale for this approach was that the hydrogen peroxide generated by GOX, upon its interaction with glucose, should lead to an electrical response of the PTEchem sensor, as established before (Fig. 6a). Notably, the GOX-derivatized PTEchem sensor responded in a dose-dependent manner to glucose concentration range of 0.25 to 10.0 mM, and demonstrated sensor reversibility response within this concentration range (Fig. 6b, left graph). The drift in the sensor’s reverse response at decreasing doses of glucose could be explained by the delayed release of the accumulated H_2_O_2_ from the hydrogel to the surrounding medium. Non-linear regression log(2) fit of the dose-dependent sensor response to glucose (Fig. 6b, right graph) shows a linear correlated fit response in physiological concentration range of 0.5–10 mM, with an LOD = 0.25 mM for glucose. The data is presented as the normalized PTEchem sensor response (relative to the PB baseline), and representative results of ten sensors, overall displaying an in vitro limit of detection (LOD) < 0.5 mM and physiologically relevant operational concentration range for glucose detection. Overall, these results indicate the potential usability of this sensor platform for continuous glucose monitoring.

**Fig. 7:**
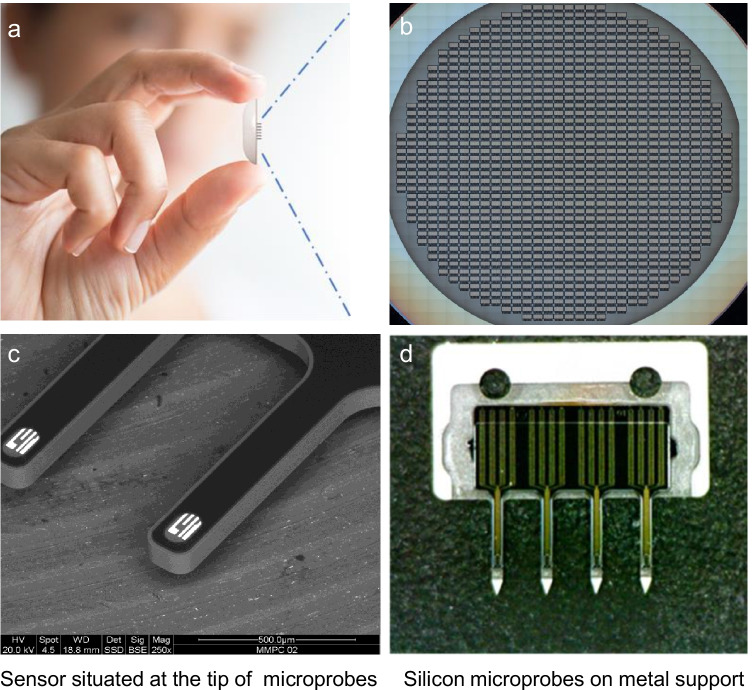
**a** Patch-assembled PTEchem sensor array. **b** Wafer-level fabrication of the PTEchem sensor. **c** Silicon microprobes carrying PTEchem sensors at their tips. **d** PTEchem sensor microprobe-based sensors laminated with a stainless steel support structure for effective skin insertion

In order to further adapt the developed PTEchem sensor technology to wearable biomedical applications, we have fabricated a small, ~ 2 × 4 mm silicon chip with ~ 1.5 mm long metal-supported microprobes, the latter serving to assist with intradermal sensor introduction and positioning (Fig. 7). The outer diameter of these intradermal microprobes is ~ 150 µm, equivalent to a 35-gauge needle, which facilitates painless and minimally invasive skin insertion. The features and dimensions of these microprobes are realized via front dry Si etch and overall wafer thinning to 70–100 µm. Essentially, the PTEchem sensor array is positioned at the tips of these microprobes, allowing their intradermal placement and direct contact with the interstitial fluid (ISF) for vital biochemical and metabolic monitoring and data collection.

## Discussion

This paper presents a new paradigm of potentiometric electrochemical sensing, which is based on the electric potential difference between the solution analyte and a polarizable electrode. Evidently, a change in the electrode’s potential, due to its interaction with a redox species in the solution, drives its polarization and the subsequent change in the current (*I*_sd_) of a coupled Fin-FET. We postulate that these changes in FET depletion/accumulation are attributed to the interaction of the redox analyte with the sensor’s WE, followed by reaching a potential equilibrium between the analyte and the WE with respect to the drain potential. We base this hypothesis on results where passivation of the entire gate oxide by PTFE/SU-8 layer distinctively limited the interaction between the analyte and the gate oxide. Accordingly, we show that the effect of a specific analyte on the depletion/accumulation state of the coupled SiF-FET is governed by the biasing potential. Specifically, a redox reaction at the WE can be either oxidation or reduction (or none), in accordance with the difference in redox potential between the analyte and the electrode voltage setting. Since the same biasing potentials will determine the entire FET operating region, the fabricated FET needs to be designed to work as close as possible to its linear region under the same electrochemical biasing conditions. To demonstrate commercial usability, the PTEchem sensor device was tested under relevant physiological conditions, at the micro- to millimolar concentration range of common redox species. Under these conditions, the electrode is polarized accordingly, which is amplified by the adjacent SiF-FET. In fact, the requirement to operate in high molarity charged environment, as occurs under physiological conditions, excludes the majority of gate oxide–based FET sensors. In contrast, the PTEchem sensor described here is not limited by these conditions and displays a dynamic range compatible with physiological glucose concentrations.

Notably, we have demonstrated that the novel sensing paradigm described in this paper can be used to discriminate between specific redox species in solution by tuning the biasing potential scheme. This analyte-specific tunability aims at achieving noise reduction and filtering, further supporting sensor implementation in wearable devices for continuous multi-metabolite monitoring. Moreover, this concept enables a dramatic reduction in sensor size and footprint, favoring its usability in minimally invasive sensors, such as those used for wearable continuous glucose monitoring (CGM) devices. Furthermore, the ability to simultaneously place multiple sensors in a miniature-sized array format opens the possibility for parallel monitoring of multiple analytes, employing microprobes for sensor separation and intradermal positioning. Multiple studies have previously demonstrated the ability to sense redox species using FET-based technologies. However, the FET-based sensors in these studies have utilized reactive gate materials, such as iridium oxide, or active redox mediators, such as HRP and Prussian blue [22, 24, 25]. Evidently, redox species sensing may also be achieved through nanowire FET surface modification of the gate material, by coupling known redox mediators such as quinone/hydroquinone [27]. However, these chemical modification schemes used for nanowire functionalization suffer from limited reversibility between the reduced and oxidized states, hindering their implementation in sensors for continuous monitoring applications.

PTEchem sensor platform described here utilizes chemically inert materials, such as gold and platinum, for sensor electrode construction, allowing continuous, fully reversible, and stable sensing of different redox species in solution. The tunable nature of the PTEchem sensor imparts it with superior analyte selectivity, allowing excellent “physiological noise” filtration. Furthermore, multi-analyte sensing can be realized by employing an array of PTEchem sensors, each voltametrically tuned to detect a different analyte. Taken together with the superior low power consumption of nanoFETs and their small footprint, this novel PTEchem sensor platform has the potential to surpass traditional amperometric sensors in multiple biological and medical diagnostic applications.

## Supplementary Information

Below is the link to the electronic supplementary material.

Supplementary file1 (PDF 861 kb)

## References

[1] Teymourian H, Barfidokht A, Wang J (2020). Electrochemical glucose sensors in diabetes management: an updated review (2010–2020). Chem Soc Rev.

[2] Schmelzeisen-Redeker G, Staib A, Strasser M, Müller U, Schoemaker M (2013). Overview of a novel sensor for continuous glucose monitoring. J Diabetes Sci Technol.

[3] Joseph JI (2021). Review of the long-term implantable senseonics continuous glucose monitoring system and other continuous glucose monitoring systems. J Diabetes Sci Technol.

[4] Pleitez MA, Lieblein T, Bauer A, Hertzberg O, von Lilienfeld-Toal H, Mäntele W (2013). In vivo noninvasive monitoring of glucose concentration in human epidermis by mid-infrared pulsed photoacoustic spectroscopy. Anal Chem.

[5] Alsunaidi B, Althobaiti M, Tamal M, Albaker W, Al-Naib I. A review of non-invasive optical systems for continuous blood glucose monitoring. Sensors. 2021;21(20). 10.3390/s21206820 PMC8537963 34696033

[6] Fagan-Murphy A, Hachoumi L, Yeoman MS, Patel BA (2016). Electrochemical sensor for the detection of multiple reactive oxygen and nitrogen species from ageing central nervous system homogenates. Mech Ageing Dev.

[7] Li Y, Hu K, Yu Y, Rotenberg SA, Amatore C, Mirkin MV (2017). Direct electrochemical measurements of reactive oxygen and nitrogen species in nontransformed and metastatic human breast cells. J Am Chem Soc.

[8] Zdrachek E, Bakker E (2019). Potentiometric sensing. Anal Chem.

[9] Walker NL, Roshkolaeva AB, Chapoval AI, Dick JE. Recent advances in potentiometric biosensing. Curr Opin Electrochem. 2021;28. 10.1016/j.coelec.2021.100735 PMC8162913 34056144

[10] Forouhi S, Ghafar-Zadeh E. Applications of CMOS devices for the diagnosis and control of infectious diseases. Micromachines (Basel). 2020;11(11). 10.3390/mi11111003 PMC7698050 33202888

[11] Bergveld P (2003). Thirty years of ISFETOLOGY: what happened in the past 30 years and what may happen in the next 30 years. Sens Actuators, B Chem.

[12] Kaisti M. Detection principles of biological and chemical FET sensors. Biosensors and Bioelectronics. 2017;98. 10.1016/j.bios.2017.07.010 28711826

[13] Janata J (2004). Thirty years of CHEMFETs – a personal view. Electroanalysis.

[14] Bergveld P (1970). Development of an ion-sensitive solid-state device for neurophysiological measurements. IEEE Trans Biomed Eng.

[15] Mele LJ, Palestri P, Selmi L (2020). General approach to model the surface charge induced by multiple surface chemical reactions in potentiometric FET sensors. IEEE Trans Electron Devices.

[16] Shalev G, Landman G, Amit I, Rosenwaks Y, Levy I (2013). Specific and label-free femtomolar biomarker detection with an electrostatically formed nanowire biosensor. NPG Asia Materials..

[17] Shinwari MW, Zhitomirsky D, Deen IA, Selvaganapathy PR, Deen MJ, Landheer D (2010). Microfabricated reference electrodes and their biosensing applications. Sensors (Basel).

[18] Hendrikse J, Olthuis W, Bergveld P. ChemInform Abstract: Characterization of the EMOSFET, a novel one-electrode chemical transducer for redox measurements. ChemInform. 1999;30(8).

[19] Hendrikse J, Olthuis W, Bergveld P (1998). The EMOSFET as a potentiometric transducer in an oxygen sensor. Sens Actuators, B Chem.

[20] Anh DTV, Olthuis W, Bergveld P (2004). Work function characterization of electroactive materials using an /sup E/MOSFET. IEEE Sens J.

[21] Lee J-Y, Lee JG, Lee S-H, Seo M, Piao L, Bae JH (2013). Hydrogen-atom-mediated electrochemistry. Nature. Communications.

[22] Anh DTV, Olthuis W, Bergveld P (2002). Electroactive gate materials for a hydrogen peroxide sensitive /sup E/MOSFET. IEEE Sens J.

[23] Heifler O, Borberg E, Harpak N, Zverzhinetsky M, Krivitsky V, Gabriel I, et al. Clinic-on-a-needle array toward future minimally invasive wearable artificial pancreas applications. ACS Nano. 2021. 10.1021/acsnano.1c03310 PMC8397432 34157222

[24] Rani D, Rollo S, Olthuis W, Krishnamoorthy S, Pascual García C. Combining chemical functionalization and FinFET geometry for field effect sensors as accessible technology to optimize pH sensing. Chemosensors. 2021;9(2).

[25] Tomari N, Sasamoto K, Sakai H, Tani T, Yamamoto Y, Nishiya Y (2019). New enzymatic assays based on the combination of signal accumulation type of ion sensitive field effect transistor (SA-ISFET) with horseradish peroxidase. Anal Biochem..

[26] Lucarelli F, Ricci F, Caprio F, Valgimigli F, Scuffi C, Moscone D (2012). GlucoMen day continuous glucose monitoring system: a screening for enzymatic and electrochemical interferents. J Diabetes Sci Technol.

[27] Krivitsky V, Zverzhinetsky M, Patolsky F (2020). Redox-reactive field-effect transistor nanodevices for the direct monitoring of small metabolites in biofluids toward implantable nanosensors arrays. ACS Nano.

