# Supplementary material for: Potentio-tunable FET sensor having a redox-polarizable single electrode for the implementation of a wearable, continuous multi-analyte monitoring device
Source: Anal Bioanal Chem. 2022 Feb 1;414(10):3267–77. doi: 10.1007/s00216-022-03911-0 (PMC8956537; doi:10.1007/s00216-022-03911-0)
Supplement: Supplementary file 1 — Supplementary file1 (PDF 861 kb) [file 216_2022_3911_MOESM1_ESM.pdf]

Electronic Supplementary Information

Potentio-Tunable FET sensor having a redox-polarizable single electrode for the implementation of a wearable, continuous multianalyte monitoring device

Sharon Lefler*[1], Berta Ben-Shachar[1], Hila Masasa*[1], David Schreiber[1] and Idan Tamir[1]

[1] Qulab Medical, Herzliya, Israel

*** Equal contribution**

Corresponding author email address: idan@qulabmedical.com

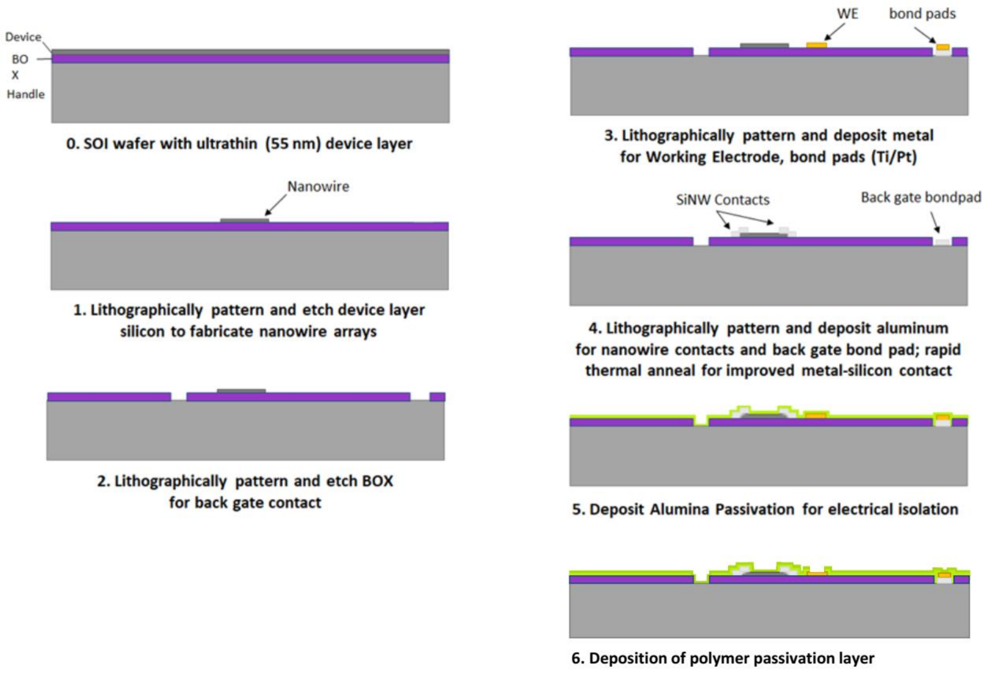

Figure S1. Key sensor fabrication steps illustration

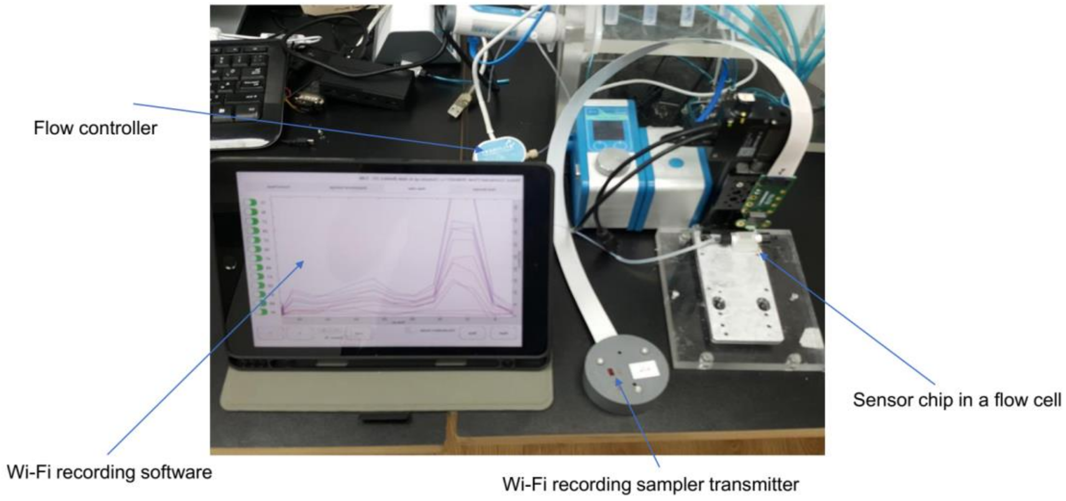

Figure S2. In-vitro sensing and fluidic systems and BT-enabled wireless transducer for continuous monitoring.

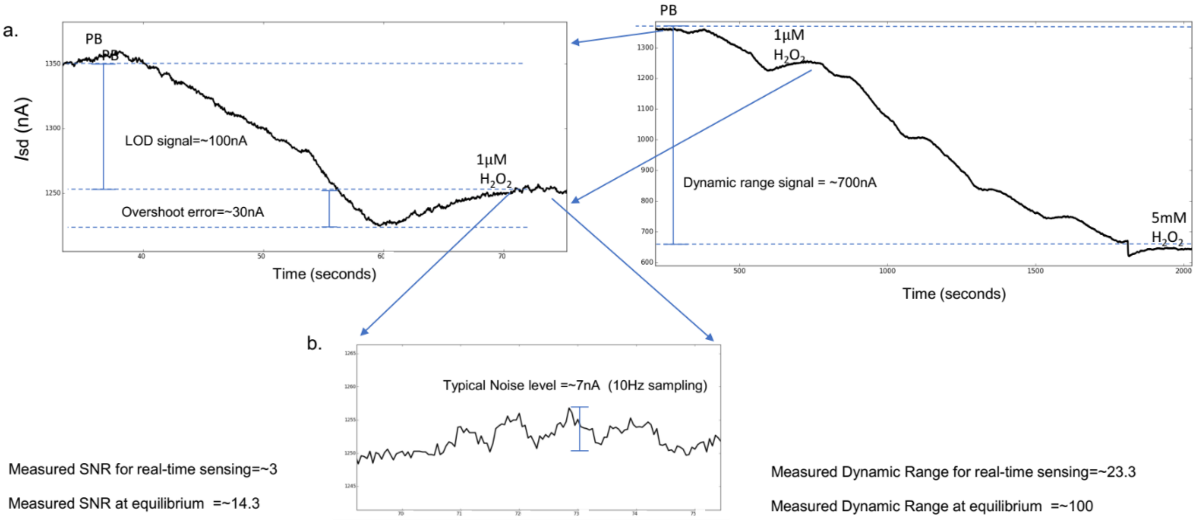

Figure S3. Measured signal to noise ratio (SNR) for real-time and equilibrium sensing. **a,** Raw data presented in figure 6a was further used to extrapolate the, Dynamic Range and LOD Signals, as well as Overshoot Error and **b,** Typical Noise level, based on which the LOD SNR and dynamic ranges were quantified as the ratio of the highest measured output of the biosensor to the lowest measured output of the biosensor, correspondingly.
